# Identification and Validation of METTL3-Related Molecules for Predicting Prognosis and Efficacy of Immunotherapy in Gastric Cancer Based on m6A Methylome and Transcriptome Sequencing Analysis

**DOI:** 10.3389/fonc.2022.935239

**Published:** 2022-07-29

**Authors:** Shuran Chen, Xu Su, Jing Wang, Ni Zheng, Yuan Tang, Guisen Peng, Rui Dong, Fei Lu, Mulin Liu, Yunli Zhao, Huazhang Wu

**Affiliations:** ^1^ Department of Gastrointestinal Surgery, Anhui Province Key Laboratory of Translational Cancer Research, First Affiliated Hospital of Bengbu Medical College, Bengbu, China; ^2^ School of Life Science, Anhui Province Key Laboratory of Translational Cancer Research, Bengbu Medical College, Bengbu, China; ^3^ School of Public Health, Bengbu Medical College, Bengbu, China

**Keywords:** METTL3, gastric cancer, m6A modification, immune microenvironment, immune checkpoint

## Abstract

Abnormal N6-methyladenosine (m6A) modification levels caused by METTL3 have been identified to be a critical regulator in human cancers, and its roles in the immune microenvironment and the relationship between targeted therapy and immunotherapy sensitivity in gastric cancer (GC) remain poorly understood. In this study, we assessed the transcriptome-wide m6A methylation profile after METTL3 overexpression by m6A sequencing and RNA sequencing in BGC-823 cells. Gene Ontology (GO) and Kyoto Encyclopedia of Genes and Genomes (KEGG) analyses were performed to analyze the function of core targets of METTL3. Eighteen methylation core molecules were identified in GC patients by combining transcriptome and methylome sequencing. GC patients can be separated into two subtypes based on the expression of 18 methylation core molecules. Furthermore, subgroup analysis showed that patients with different subtypes had a different OS, PFS, stage, grade, and TMB. Gene set enrichment analysis (GSEA) showed that immune-related pathways were enriched among subtype A. The ESTIMATE analysis suggested that the extent of infiltration of immune cells was different in two subtypes of GC patients. Tumor Immune Dysfunction and Exclusion (TIDE) and The Cancer Immunome Atlas (TCIA) database also showed that there were significant differences in the efficacy of immunotherapy among different types of GC patients. Altogether, our results reveal that METTL3-mediated m6A methylation modification is associated with the immune microenvironment and the effects of immunotherapy in GC patients. Our findings provide novel insights for clinicians in the diagnosis and optimal treatment of GC patients.

## Introduction

With changes in dietary and lifestyle habits, the incidence and mortality of gastric cancer (GC) have increased substantially over the past decade, and reports suggest that the median survival time of advanced GC is only 12–15 months (1, 2). The Global Cancer statistics 2020 report shows that the incidence and mortality of GC ranked fifth and fourth, respectively, among the malignant tumors (3). Excitingly, immune checkpoint inhibitors have made great strides in treating advanced GC in recent years. It has been shown that the combination of PDL1 inhibitors in chemotherapy and targeted therapy can significantly reduce tumor size and improve objective response rates (4, 5). However, some GC patients do not derive benefits from immunotherapy (6).

N6-methyladenosine (m6A) is the most abundant RNA base modification in mammalian mRNAs, especially in eukaryotic mRNA (7), and METTL3 is the core methyltransferase of m6A modification, which plays a critical biological role in the occurrence and development of various malignant tumors by regulating gene expression. Numerous studies have demonstrated that METTL3 can facilitate the growth of GC cells and liver metastasis by promoting angiogenesis and glycolysis (8). METTL3 also mediates the repression of E-cadherin by enhancing m6A modification of ZMYM1, thereby promoting the epithelial–mesenchymal transition of GC cells (9). Moreover, METTL3 can influence the immune microenvironment in tumors and thus affect the sensitivity of patients to immunotherapy (10). In addition, decreased METTL3 expression in bone marrow cells can promote tumor growth and metastasis *in vivo*, and the infiltration of M1 and M2 tumor-associated macrophages and regulatory T cells was increased significantly in tumor tissues in METTL3-deficient mice (11). Meanwhile, METTL3 can upregulate PD-L1 expression and inhibition of METTL3 expression can enhance tumor immunity through PD-L1-mediated T-cell activation *in vitro* and *in vivo* (10). However, the effect of METTL3-induced m6A modification on the GC immune microenvironment has not been thoroughly studied. Therefore, a comprehensive understanding of genes regulated by METTL3 in GC and the relationship between these molecules and the immune microenvironment of GC may provide a foothold for understanding the pathogenesis of GC and predicting the immune response of patients to GC.

## Materials and methods

### Cell Culture

The human GC cell line BGC-823 was purchased from the Institute of Cell Research of the Chinese Academy of Sciences in Shanghai. The cells were cultured in DMEM (Procell) containing 10% fetal bovine serum (FBS; Gibco) and 1% penicillin–streptomycin (Biyuntian) in a 37°C incubator with 5% CO_2_, and sterile water was added to maintain humidity in the incubator.

### Establishment of Stable Cell Lines Overexpression METTL3

The METTL3-overexpressing lentiviral fluid was obtained from Jikai (Shanghai Jikai Gene Medical Technology Co., Ltd. Shanghai, China). BGC-823 cells in the logarithmic growth phase were inoculated into a 6-well plate, when the confluence was close to 70%, and the negative control (NC group) and METTL3-overexpressing lentivirus (oe-METTL3) were used to infect BGC-823 cells according to the manufacturer’s instructions. Following lentiviral infection, puromycin (4 μg/ml) was used for screening for 3 days, and the successfully transfected cells were used for subsequent experiments.

### Transcriptome and Methylome Sequencing

Total RNA isolated from BGC-823 cells transfected with control and METTL3-overexpressing lentivirus was subjected to m6A-mRNA and lncRNA. Epitranscriptomic microarray and transcriptome sequencing were performed by Aksomics Inc. (Shanghai, China) and Beijing Genomics Institute (BGI, China), respectively. Agilent Feature Extraction software (version 11.0.1.1) analyzed the acquired array images. Raw intensities of IP (immunoprecipitated, Cy5-labeled) and Sup (supernatant, Cy3-labeled) were normalized with an average of log2-scaled spike-in RNA intensities. After spike-in normalization, the probe signals with Present (P) or Marginal (M) QC flags in a certain proportion were retained for m6A quantification based on the IP (Cy5-labeled) normalized intensities. Differentially m6A-methylated RNAs between two comparison groups were identified using the screening criteria for the fold change and statistical significance (*p*-value) thresholds. Hierarchical clustering was performed to show the differential m6A-methylation patterns among samples.

### Screening of Candidate Methylated Key/Core Genes and Functional Enrichment Analysis

Differentially expressed genes with low methylation degree and increased expression were selected using screening criteria *p*-value < 0.05 and fold-change ≤ 1/1.5, and genes with high methylation and decreased expression were screened with *p*-value < 0.05 and fold-change ≥ 1.5. The intersection of genes with high methylation and decreased expression and genes with low methylation degree and increased expression yielded the methylation core genes. Functional enrichment analysis of differential genes was performed using the Database for Annotation, Visualization and Integrated Discovery (DAVID) database (https://david.ncifcrf.gov/), and the enrichment results were visualized using the “ggplot2” package.

### Data Collection

Clinical information, comprehensive transcriptome, and mutational profiling of 804 GC patients were obtained from the GSE84437 dataset of the TCGA and GEO databases, and the data were corrected using the “limma” package.

### Consensus Clustering

GC patients were divided into different molecular subtypes based on the expression levels of core genes regulated by METTL3 using “ConsensusClusterPlus”. Good clustering was based on the following requirements: (1) the cumulative distribution function curve increases steadily; (2) the sample distribution of each group is relatively even, and there is no maximal or minimal grouping; and (3) the correlation of the intergroup is low, and that of the intragroup is high.

### Assessment of Tumor Mutational Burden, Immune Microenvironment, Immune Responses, and Drug Treatment in Different GC Subtypes

The mutational data of GC patients were downloaded from the TCGA database and processed by Pl script. “ggplot2” and “survival” package were used to draw the Kaplan–Meier (K-M) survival curves and violin plot of overall survival (OS) and tumor mutational burden (TMB) in different GC subtypes. The scores of stromal cells, immune cells, and total tumor cells were calculated separately using the ESTIMATE algorithm (12). The contents of various types of immune cells in GC tissues were analyzed using CIBERSORT (13). Immune checkpoint genes and immunophenotyping data were obtained from previous studies (14, 15). Immunotherapy data for GC patients were obtained from TIDE (http://tide.dfci.harvard.edu/) and TCIA (https://tcia.at/) websites. The “pRRophetic” package was used to predict the sensitivity of different GC subtypes to clinical drug treatment.

## Results

### Genome-Wide Screening of Altered m6A-Tagged Transcript Profiles in GC Cells After METTL3 Overexpression

**Figure 1:**
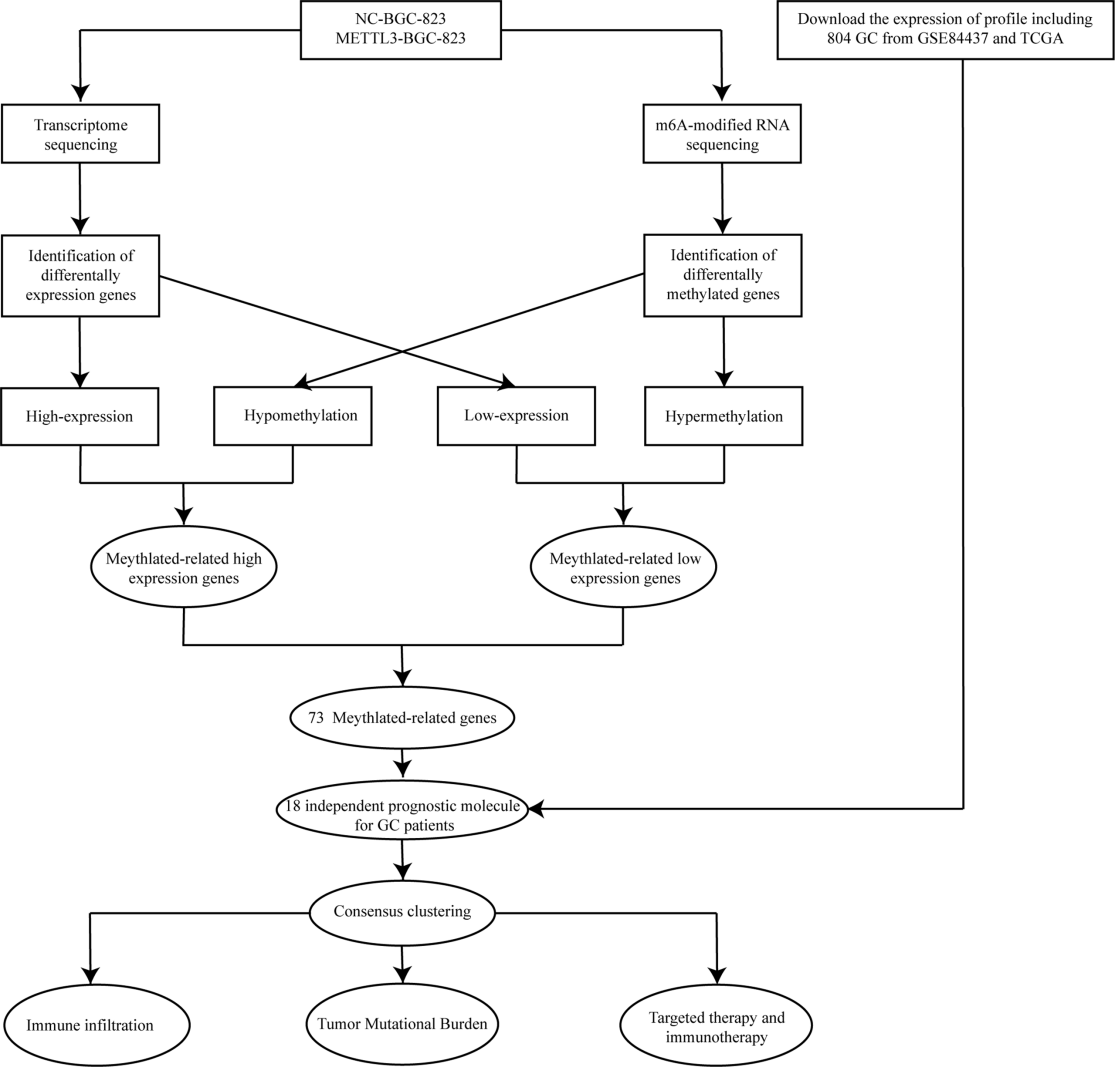
The flowchart showing the design of this study.

**Figure 2:**
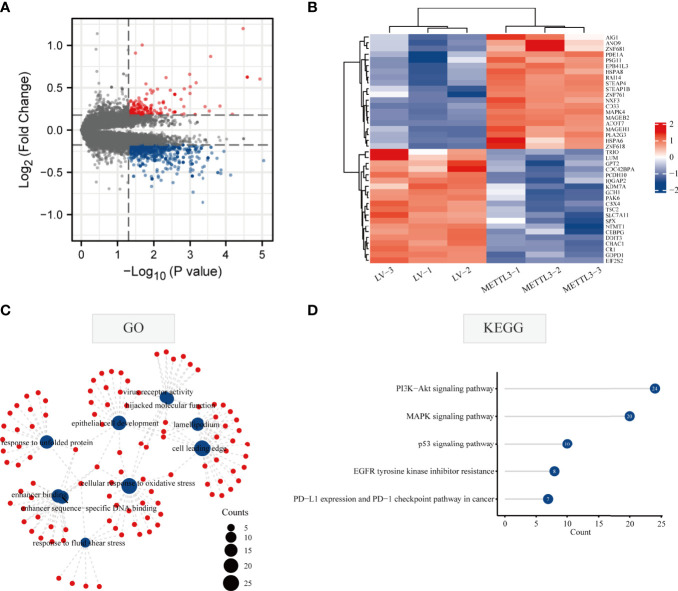
Methylation modification of METTL3 regulatory targets. **(A)** Volcano plot of the methylation level of differential genes regulated by METTL3. The red and blue dots in the plot represent high and low m6A methylation modification genes, respectively. Black spots represent genes with no difference in methylation levels regulated by METTL3. **(B)** The top 20 genes with the highest or lowest difference in methylation levels regulated byMETTL3 were shown in the heatmap. **(C, D)** GO and KEGG enrichment analysis of genes with different methylation levels.

The flowchart of the study is indicated in **Figure 1**. To systematically explore the RNA m6A modification profile after METTL3 overexpression, genome-wide profiling of m6A-tagged transcripts was conducted by m6A-modified RNA sequencing (m6A-seq). The results showed that overexpression of METTL3 resulted in significantly increased and decreased m6A modifications in 150 and 525 genes, respectively. A total of 28 hypomethylated, highly expressed genes were identified. Next, a volcano plot was generated from the above differentially methylated genes to visualize the differentially expressed genes. Then, a heatmap was generated to visualize the top twenty genes with the highest or lowest methylation levels (**Figures 2A, B**). Finally, GO and KEGG enrichment analyses were performed on differentially methylated genes, and the results showed that m6A-methylated genes affected by METTL3 were mainly enriched in PI3K-AKT, MAPK, and P53 signaling pathways. Interestingly, these genes were also associated with tyrosine kinase inhibitors and PD-L1 (**Figures 2C, D**; **Supplementary Table 1**). These results indicated that m^6^A modification changes caused by METTL3 might be promising targets for immunotherapy.

### Effects of METTL3 Gene Overexpression on the Transcriptome Profiling of GC Cells

**Figure 3:**
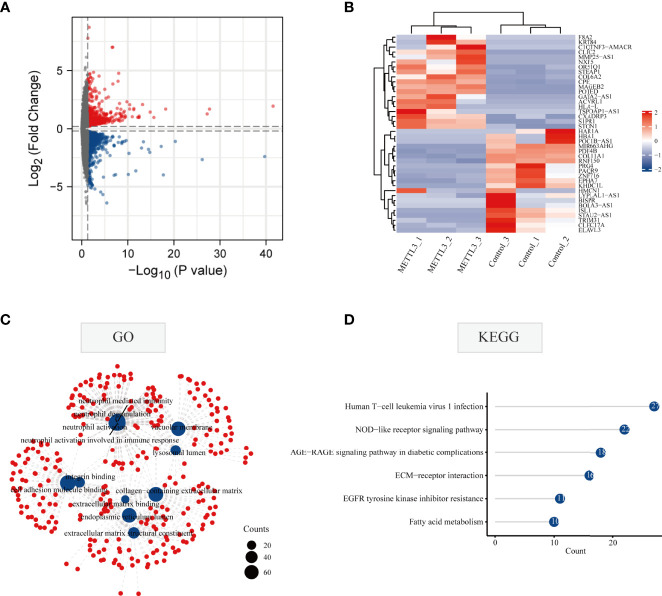
Gene alterations at the transcriptional level regulated by METTL3 overexpression. **(A)** Volcano diagram of differentially expressed genes regulated by METTL3. The red and blue dots in the plot represent upregulated and downregulated genes, respectively. Black spots represent genes changed with no significance. **(B)** The top 20 genes with the highest or lowest change of expression regulated by METTL3 were shown in the heatmap. **(C)** GO enrichment analysis of METTL3-regulated differentially expressed genes at the transcriptional level; **(D)** KEGG enrichment analysis of METTL3-regulated differentially expressed genes at the transcriptional level.

To explore the effect of METTL3 on gene expression at the transcriptome level, the genes and signaling pathways that METTL3 may regulate were analyzed by transcriptome sequencing in METTL3-overexpressed BGC-823 cells. A total of 1,266 upregulated genes and 1,526 downregulated genes were yielded by transcriptome sequencing. First, all differentially expressed genes were visualized using volcano plot representations, followed by a heatmap to visualize the top twenty genes with the most increased or decreased expression level influenced by METTL3 (**Figures 3A, B**). Next, GO and KEGG enrichment analyses were performed on the differentially expressed genes, and the results showed that these genes were mainly related to cell adhesion. Similar to m6A RNA methylomes revealed by m6A-seq, differential genes in the transcriptome were enriched significantly in immune and tyrosine kinase inhibitor resistance pathways (**Figures 3C, D**; **Supplementary Table 2**). The above results indicate that METTL3 participates in signal transduction pathways involved in tumorigenesis and development, and may also be closely related to the clinical targeting of GC and immunotherapy sensitivity.

### Identifying Differences in m6A-Methylated Core Genes Regulated by METTL3 in GC Cells

**Figure 4:**
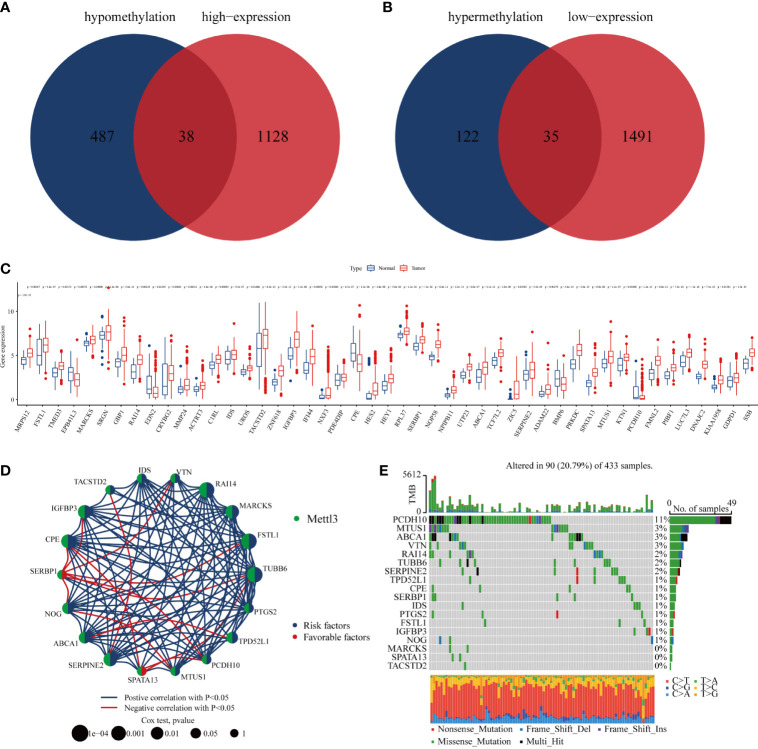
Core genes regulated by METTL3. **(A, B)** Venn plot of combined transcriptome and epigenome sequencing of core genes; **(C)** differential expression of core genes in gastric cancer and normal gastric mucosal tissues; **(D)** core genes with independent prognostic value identified by Cox regression; **(E)** mutation rate of core genes with independent prognostic value in gastric cancer patients.

Previous studies have confirmed that m6A modification can affect the stability of RNA (16). Accordingly, we first screened the genes with high m6A methylation but low expression, and then intersected them with genes with low m6A methylation and increased expression; 73 genes were selected for further exploration (**Figures 4A, B**). The results showed that most methylated core genes were differentially expressed in GC tissues compared with normal tissues (**Figure 4C**). Survival analysis by Cox regression and K-M analyses indicated that 18 methylation core molecules had prognostic value for GC patients (**Figure 4D**; **Supplementary Table 3**). Then, the relationship between the mutation status of methylation core genes and the prognosis of patients with GC was analyzed; the waterfall plot showed that 11% of GC patients had mutations in the PCDH10 gene, and the rest of the methylation core genes also had different degrees of mutation (**Figure 4E**). In conclusion, we identified core genes that were methylated by METTL3, which may play important roles in the progression of GC.

### Molecular Subtype Analysis of Methylated Core Genes Regulated by METTL3

**Figure 5:**
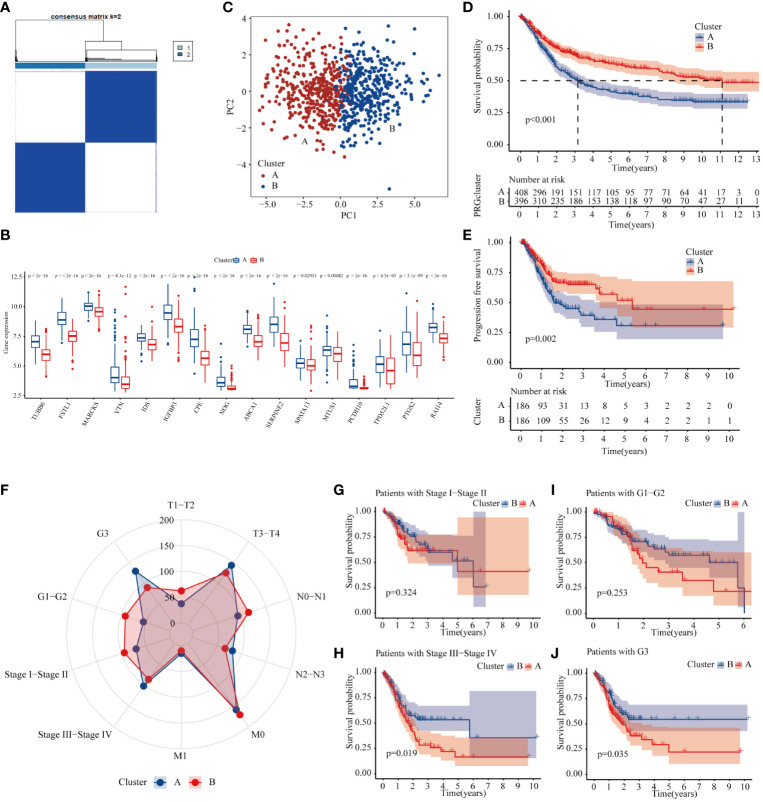
Molecular subtype of METTL3-regulated core genes. **(A)** Consensus clustering of gastric cancer patients according to the expression of core genes. **(B)** The expression of 18 methylation core molecules in different GC subtypes. **(C)** PCA plots of subtype. **(D, E)** K-M curves of overall survival and progression-free survival of gastric cancer patients with different subtypes. **(F)** The number of patients at different T, N, and M stages and grades in different GC subtypes. **(G–J)** The prognosis of Stage I–Stage IV and G1–G3 GC patients in different GC subtypes.

To explore the effect of METTL3-mediated m6A methylation on core genes in GC progression (**Supplementary Table 4**), a consensus clustering algorithm was applied to classify GC patients into two clusters based on the expression levels of 18 methylation core molecules (**Figure 5A**). The histogram showed that these genes were overexpressed mainly in type A (**Figure 5B**). The principal component analysis (PCA) plot showed a significant difference between the two GC subtypes (**Figure 5C**). Subsequently, we analyzed the effect of each subtype on GC patient prognosis. The K-M curve showed that the OS and progression-free survival of Cluster B GC patients were markedly better than those of Cluster A (**Figures 5D, E**). Finally, we investigated the association between clinicopathologic features and different GC types. The radar chart displays GC patients in T3–T4, N2–N3, M1, Stage III–Stage IV, and G3, and is clearly concentrated in the A subtype (**Figure 5F**). Meanwhile, the prognosis of patients in the B subtype with Stage III–Stage IV was significantly better than GC patients in the A subtype (**Figures 5G–J**). In summary, core genes methylated by METTL3 may be a marker of prognosis for patients with GC.

### Tumor Mutational Burden and Immune Microenvironment are Significantly Different Among GC Patients with Different Molecular Subtypes

**Figure 6:**
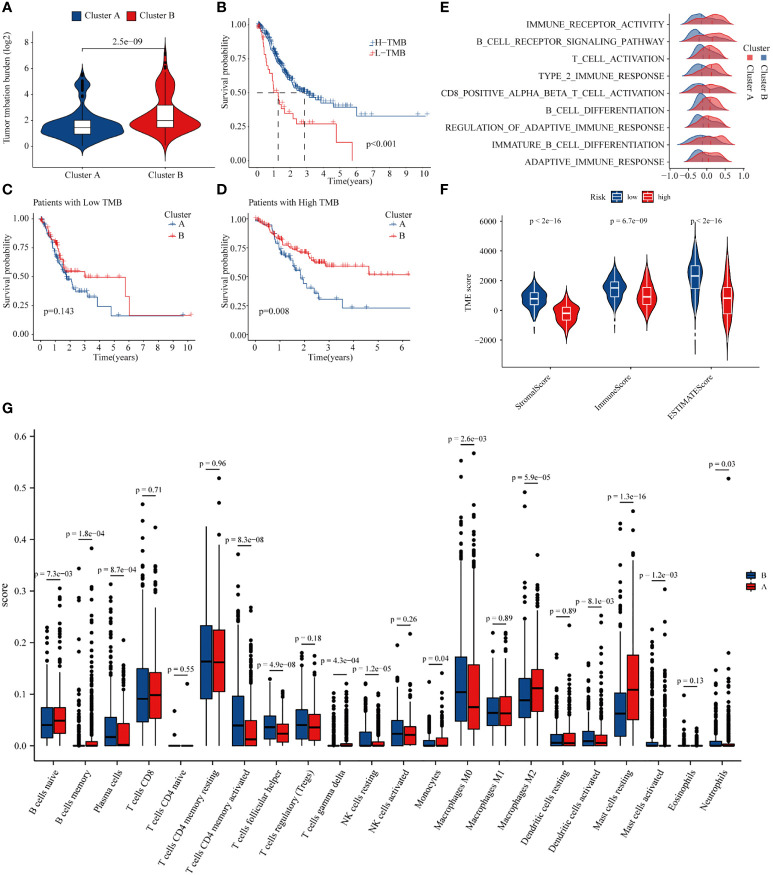
Differences in tumor mutational burden and immune microenvironment in gastric cancer patients in different subtypes. **(A)** Tumor mutational burden of different GC subtypes. **(B)** Relationship between tumor mutational burden and prognosis of GC patients. **(C, D)** Prognosis of patients of high- and low-TMB in A or B GC subtpyes. **(E)** GSEA enrichment analysis of subtype. **(F)** Differences in the immune microenvironment in gastric cancer patients in different subtypes. **(G)** Differences in immune cell infiltration in different subtypes.

It is widely acknowledged that the TMB is closely related to the degree of response of patients to treatment (17). Accordingly, we explored the differences in TMB among molecular subtypes of GC patients. The results indicated that patients with B molecular subtypes had a considerably higher TMB (**Figure 6A**). Subsequently, we combined different molecular subtypes and TMBs to analyze GC prognosis. The results showed that the prognosis of GC patients with a high TMB was significantly better than those with a low TMB, and the prognosis of patients with a high TMB in the B subtype was significantly better than type A patients (**Figures 6B–D**). Since TMB is closely related to the immunity of GC patients, we used GSEA to explore the differences in immune-related functions between the two subtypes. The results showed that most immune-related biological processes and molecular functions, such as immune receptor activity, T-cell activity, and B-cell differentiation, were significantly different between the two types (**Figure 6E**; **Supplementary Table 5**). We then analyzed the differences in immune microenvironment scores between the two subtypes. Interestingly, subtype B had significantly lower stromal and immune scores than subtype A (**Figure 6F**). Finally, based on CIBERSORT, we explored the differences in immune cell infiltration in GC patients with different subtypes. The infiltration levels of M2 macrophages, monocytes, resting mast cells, CD8 T cells, and naïve B cells were obviously higher in subtype A than those in subtype B, while follicular helper T cells, memory-activated CD4 T cells, and M0 macrophages had significantly lower infiltration in subtype A compared to those in subtype B (**Figure 6G**). Altogether, significant disparity of TMB and immune cell infiltration was found between the different subtypes of GC based on 18 methylation core molecules.

### Analysis of Differences in Immune and Targeted Therapy Responsiveness in Gastric Cancer Patients of Different Molecular Subtypes

**Figure 7:**
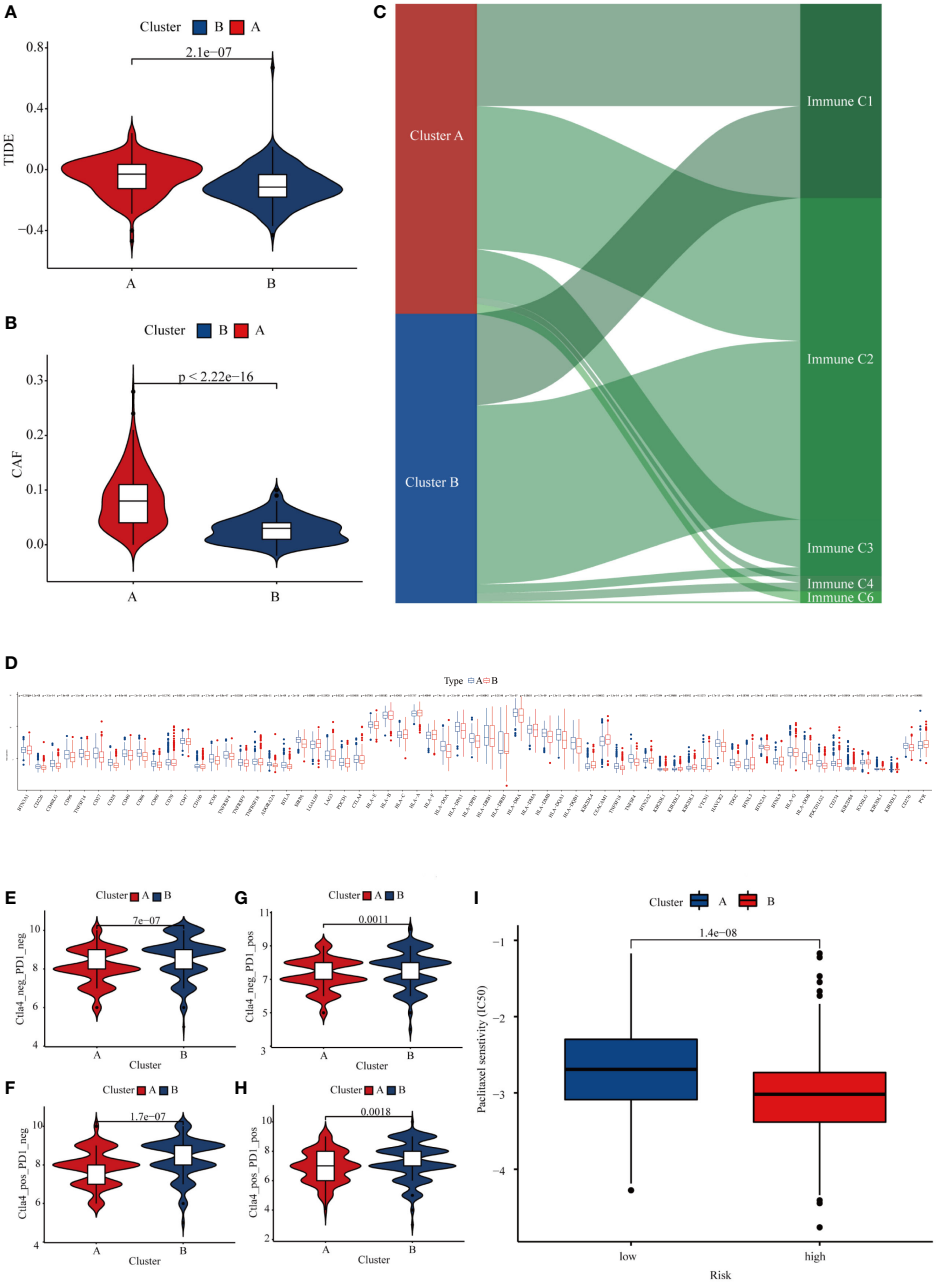
Differences in the responsiveness of patients to chemotherapy, targeted therapy, and immunotherapy in different GC subtypes. **(A)** The TIDE score in different GC subtypes. **(B)** Expression of CAF in different GC subtypes. **(C)** The relationship between GC subtypes and immune expression signatures. **(D)** Expression of immune checkpoints in different GC subtypes. **(E–H)** Differences in the responsiveness of GC patients to immune checkpoint inhibitor therapy in different subtypes. **(I)** The sensitivity of paclitaxel in different GC subtypes.

An increasing body of evidence suggests considerable heterogeneity in the efficacy of immunotherapy in patients with GC, and TMB is a biomarker that can predict the response or efficacy of immunotherapy in GC patients (18). We used the Tumor Immune Dysfunction and Exclusion (TIDE) score to explore differences in immune responses between the two subtypes. Subtype B had a relatively low TIDE score, suggesting that subtype B of GC patients may have a better response to immunotherapy (**Figure 7A**). It is well-established that CAF is involved in the body’s immune response (19). Our analysis showed that GC patients with subtype B have significantly lower CAF levels, which may account for subtype B patients deriving greater benefit from immunotherapy (**Figure 7B**). In addition, we explored the relationship between GC subtypes and immune expression signatures, and we found that subtype A patients have a greater proportion of C4 and C6 (**Figure 7C**). Because GC patients with subtype B have a higher TMB, we explored whether patients with subtype B have a higher sensitivity to immune checkpoint inhibitors. First, we found that 42 immune checkpoint genes were differentially expressed in the two GC subtypes, including LAG3, CD274, PDCD1, and CTLA-4 (**Figure 7D**). We then obtained the immunotherapy score data from TCIA and compared the difference in immunotherapy score between two GC subtypes. Results demonstrated that patients with subtype B exhibited higher immunotherapy scores than subtype A (**Figures 7E–H**). Finally, we explored the differences between the two subtypes for other pharmacological treatments. The results showed that patients in subtype B exhibited higher sensitivity to paclitaxel than in type A (**Figure 7I**). Taken together, our results indicate that METTL3-mediated m6A methylation may affect the responsiveness to immunotherapy in GC patients.

## Discussion

GC is one of the leading causes of cancer-related death worldwide, especially in China. Significant inroads have been achieved in recent years; for instance, neoadjuvant chemotherapy can greatly improve the prognosis of GC patients, and GC survival rates at 5 years are less than 30% (20, 21). In recent years, tumor immunotherapy has made a breakthrough in clinical cancer treatment, but some patients still do not benefit from immunotherapy (22). Targeted epigenetic therapies can improve the efficacy of immunotherapy (23). Recent studies have found that mRNA m6A is involved in anti-inflammatory and anti-tumor functions, playing a vital role in regulating the complexity and diversity of the immune microenvironment. Accordingly, evaluating the modification pattern of m6A can help predict immunotherapy responsiveness. It is well-established that METTL3-mediated m6A modifications influence the immune microenvironment and thus affect the sensitivity of patients to immunotherapy (24). In this study, 18 methylation core molecules regulated by METTL3 were screened by using methylation and transcriptome sequencing. We found that METTL3-mediated methylation is essential for shaping the immune microenvironment, which may predict patient responsiveness to immunotherapy.

Moreover, we divided the GC patients into two subtypes based on the expression of 18 methylation core molecules; GC patients with subtype A exhibited a worse OS and progression-free survival than patients with subtype B. In addition, consistent with previous studies in bladder, cervical, ovarian, and endometrial cancers, we found that GC patients with high TMB had better OS (25). Multiple biomarkers, especially microsatellite instability and TMB, have important implications for identifying or screening patients most likely to benefit from immunotherapy (26). A high mutational burden in lung and melanoma patients is associated with a significantly higher response to immunotherapy (27, 28). In addition, GC patients with a higher mutational burden achieved longer OS when treated with topalimumab (18). Our analysis showed that GC patients with subtype B had a higher TMB and a better prognosis, indicating that patients with subtype B would benefit more from immunotherapy.

Tumor patients with a high TMB have higher levels of tumor neoantigens, which may cause changes in the function and status of immune cells in patients, resulting in more potent killing effects on tumor cells (29). The present study used the ESTIMATE algorithm to explore differences in immune cells and stromal cells in patients with different subtypes of GC (12). We found that monocytes, M2 macrophages, tumor-associated fibroblasts, CD8 T cells, and mast cells were significantly lower in subtype B than in subtype A in GC patients. Monocytes can influence the immune microenvironment and promote GC’s malignant progression (30). M2 macrophages can activate pro-angiogenic and immunosuppressive signals in tumors, especially in diffuse GC (31). Previous studies have shown that tumor-associated fibroblasts can promote the malignant progression of GC through a variety of pathways, including promoting drug resistance of GC cells, and promoting the formation of an aggressive phenotype such as proliferation, invasion, and migration in GC cells (32, 33). In addition, inhibition of mast cells may improve the therapeutic effect of immune checkpoint inhibitors (34). Meanwhile, M0 macrophages were significantly higher in subtype B patients. Previous research suggests that newly tumor-infiltrated naive M0 macrophages play anti-tumorigenic activities *via* release of TNF-α secretion (35). In addition, follicular helper T cells and memory-activated CD4 T cells were higher in subtype B patients too. According to the literature, helper T cells are necessary to activate naive B cells. Some activated naive B cells become memory cells that provide protection for the body (36). Other studies have shown that a higher CD4/CD8 ratio of pleural effusion predicts better survival for lung cancer patients receiving immune checkpoint inhibitors (37). The low infiltration of CD8 T cells and the high infiltration of memory-activated CD4 T cells in type B GC cancer may increase the efficacy of immune checkpoint inhibitor therapy in GC patients. Decreased tumor purity also plays a vital role in the malignant progression of GC, treatment resistance, and prognostic assessment (38). These differences in the infiltration of immune cells may be related to the differences in the prognosis and clinical pathological stages of the patients with different subtypes. We found that the immune score and stromal score in subtype B GC patients were lower than in patients with subtype A, which indicated that patients with subtype A had lower tumor purity. The above results reveal that the better prognosis of patients with subtype B may be due to the higher tumor purity and reduced infiltration of immune cells that promote tumor progression.

Current evidence suggests that the application of immune checkpoint inhibitors such as PD-1/PD-L1 and CTLA-4 and the combination of immunization and standard chemotherapy significantly prolong the survival of tumor patients (39). We found that type B GC patients responded better to immune checkpoint inhibitor therapy and lower interferon levels. Interferon can promote T-cell exhaustion through PDL1 (40). Accordingly, patients with type B may benefit from immune checkpoint inhibitor therapy. The results of the TIDE database also substantiated our above findings. In addition to immunotherapy, we also analyzed the differences in the sensitivity to chemotherapy and targeted drugs in GC patients, and the results showed that GC patients with subtype B were more sensitive to paclitaxel than subtype A.

In conclusion, we developed a network-based approach to investigate the m6A-driven genes regulated by METTL3 and identified the core genes based on transcriptome and methylome sequencing. These molecules are closely related to the prognosis of GC patients, the immune microenvironment, and clinical treatment sensitivity. Therefore, these findings provided novel information regarding m6A modification changes modulated by METTL3 in GC, suggesting that METTL3-related molecules may serve as a clinical target for GC patients.

## Data availability statement

The original contributions presented in the study are included in the article/**Supplementary Material**. Further inquiries can be directed to the corresponding authors.

## Author contributions

(I) Conception and design: SC, ML, HW, and YZ. (II) Administrative support: None. (III) Provision of study materials: XS, JW and FL. (IV) Collection and assembly of data: NZ and YT. (V) Data analysis and interpretation: GP and RD. (VI) Manuscript writing: SC. (VII) Final approval of manuscript: All authors.

## Funding

This work was supported by the Natural Science Foundation of Anhui Province (1908085MH257, 2108085MH291), the Natural Science in Higher Education of Anhui Province (KJ2021A0787, KJ2021A0737, KJ2021ZD0092), and the Research and Innovation Team of Bengbu Medical College (BYKC201909).

## Conflict of Interest

The authors declare that the research was conducted in the absence of any commercial or financial relationships that could be construed as a potential conflict of interest.

## Publisher’s Note

All claims expressed in this article are solely those of the authors and do not necessarily represent those of their affiliated organizations, or those of the publisher, the editors and the reviewers. Any product that may be evaluated in this article, or claim that may be made by its manufacturer, is not guaranteed or endorsed by the publisher.
